# Seed-Borne Fungi Associated with Diverse Rice Varieties Cultivated in the Western North Region of Ghana

**DOI:** 10.1155/2023/8690464

**Published:** 2023-01-27

**Authors:** Francis Mensah Ackaah, Seloame Tatu Nyaku, Edmund Darkwa

**Affiliations:** ^1^West Africa Center for Crop Improvement (WACCI), University of Ghana, PMB 30, Legon, Accra, Ghana; ^2^Department of Crop Science, School of Agriculture, College of Basic and Applied Sciences, University of Ghana, P.O. Box LG44, Legon, Accra, Ghana

## Abstract

Rice is a major staple in the Ghanaian diet. However, its production is constrained by fungal diseases. A survey was conducted in 2018 in three selected districts in the Western North Region of Ghana using a structured questionnaire and face-to-face interaction with 230 farmers to assess their knowledge, perceptions of seed-borne fungal diseases, and management practices. Additionally, fungi associated with farmer's seeds were isolated and identified through the Agar and Blotter tests. Findings indicate that 72.7% of the farmers in the selected districts relied on their saved seeds for planting. Thirteen fungal genera were associated with the rice seed samples collected from the three districts. The Juaboso district had the majority (13) of seed-borne fungi. The seed samples were categorized into various forms of discolouration, and significant differences (*P* < 0.05) existed among the seed samples for this parameter. The AGRA rice, a farmer-saved seed from Juaboso, had the highest level of seed discolouration (41.96%). Fungi identified to be associated with the dark brown/brown discolouration of rice seeds were *Bipolaris* spp., *Fusarium* spp., *Macrophomina phaseolina* and *Aspergillus* spp. The only fungus associated with the yellow/pale yellow colour was *Bipolaris* spp. The fungi *Bipolaris* spp., *Curvularia* spp., and *Botryodiplodia* spp. were associated with the dark spot discolouration. *Alternaria* spp., and *Aspergillus* spp. were observed on the greyish white seed discolouration sample. Fungi are associated with rice cultivation and vary according to district and rice variety. A complex of pathogenic and saprophytic fungi therefore infects rice grains both in field and storage conditions.

## 1. Introduction

Rice is an important food source for more than half of the world's population. About 480 million metric tons of milled rice are produced worldwide each year [1]. Consumption of rice is rising swiftly over any other produce in Africa because it is seen as a suitable food by the growing urban population [1]. Rice production area harvested and yield were 973,000 tonnes, 331,471 ha, and 29,354 hg/ha for the year 2020 [2]. Ghana spends about $450 million on rice imports to make up for the shortfall in supply [3]. The husk of rice can be used to feed animals as well as used as a source of fuel and in biochar preparation. In developing countries, rice serves as the most important crop and plays a major role in poverty alleviation and food security attainment [4]. Rice is therefore a critical component of food security in the twenty-first century for many nations [5].

Rice suffers from several biotic and abiotic agents, resulting in heavy losses to farmers. Among the several constraints to rice production, diseases caused by fungi, nematodes, and bacteria cause major economic losses [6–8]. Fungi play significant roles in reducing the quality of rice seed through their infections [4]. These pathogens cause seed discolouration, seed rot, reduced seed germination, and vigour in seedlings, as well as making the plant weak during its early growth period. Fungal diseases that are seed-borne are comparatively challenging to manage because the fungal hyphae get established and becomes dormant [9]. Most crop diseases that are important economically are seed-borne and seed transmitted, including blast disease of rice, bakanae, loose smut, flag smut, Karnal bunt, and ear cockle of wheat [10].

Worldwide, about 56 fungal pathogens are reported to infest rice, of which 41 are reported to be seed-borne [11]. Seed-borne infections in rice cause 50–80% yield losses, depending on the agroecology, disease severity, and crop susceptibility [12]. In the Ashanti Region of Ghana, about 20 fungal species (16 pathogens and 4 saprophytes) associated with rice seeds have been identified [13]. The most common fungal species recognized included *Bipolaris oryzae*, *Fusarium verticillioides*, *Fusarium oxysporum*, *Cercospora* sp., *and Curvularia lunata* [13, 14]. Farmers who rely on their farm-saved seeds are likely to contaminate their subsequent seeds for the next planting season in instances where there is seed-borne pathogen transmission from the soil.

This research was carried out to assess farmers' knowledge and their perceptions on fungal diseases associated with rice, and also isolate and identify fungal diseases associated with rice production in the Western North Region of Ghana.

## 2. Materials and Methods

### 2.1. Field Survey: Assessing Farmers Knowledge on the Fungal Seed-Borne Diseases of Rice

A field survey was conducted in the selected districts within the Western North Region, one of the newly established regions in Ghana. The selected districts were Sefwi Waiwso Municipal, Juaboso District, and Bodi District. The districts were selected based on previous records within the districts as hotspots for some seed-borne diseases, namely, “bakanae,” “foot rot,” and “blast [15]. The selected communities were also based on their popularity for the production of rice within the districts. These were Bodi (N6.261961 W-2.754935), Afere (N6.315215 W-2.813313), Amoaya, Bokabo, and Asuopiri (N6.303535 W-794272) for Bodi district; Boako (N6.394969 W-2.577142), Asafo (N6.386971 W-2.654035), Datano (N6.244280 W-2.489403), Afrimkrom (N6.382248 W-2.639918) and Gyampokrom (N6.391655 W-2.615335) for Waiwso Municipal and Juaboso (N6.344330 W-2.828550), Nkwanta (N6.381392 W-2.840373), Sanyerano, Benchema and Proso-Kofikrom for Juaboso District.

Between October and December, 2018, a field survey was conducted using a structured questionnaire to gather information from the farmers (Supplementary Method). Farmers were selected randomly as respondents. The information gathered included demographics about the farmers, sources of seeds, production, varieties, disease incidence, and control, as well as their knowledge on fungal seed-borne diseases. A printed picture plate of diseases of rice were used to aid the farmers identify and compare symptoms they observed on their various farms. A sample size of 230 farmers was selected at random for the survey (https://wacci.ug.edu.gh/content/francis-mensah-ackaah) [16]. The respondents consisted of 86, 74, and 70 for Bodi district, Juaboso district, and Wiawso municipal, respectively.

### 2.2. Seed Sample Collection

Twelve samples were collected, comprising farmer-saved seeds and certified seeds, between November and December, 2018. The samples comprised three certified seed samples, eight (8) farmer-saved seed (FSS) samples, and one (1) local seed grower seed sample. The samples were made up of the following varieties: AGRA rice, Bosome mmienu, Kotoko, Mercy, Lapete, and Agya Amoah rice (Table 1). The farmer-saved seed samples of the AGRA rice were collected from three selected localities within a district and bulked as the farmers-saved seed sample from that particular district. Samples of seeds collected from the various districts were placed into zip-lock bags, labelled, sealed, and sent to West Africa Centre for Crop Improvement (WACCI) seed laboratory and the Plant Pathology Laboratory at the University of Ghana for seed health tests and pathogen isolation and identification, respectively.

### 2.3. Physical Quality Assessment

Physical quality assessment was undertaken to separate the pure seeds from the other foreign materials, e.g., weed seeds, stones, etc. through physical examination. Samples were thoroughly mixed, and 500 g of each sample was weighed for the purity, test which was done with the use of a diaphanoscope (Ultra Swift Lite System, China). The samples examined were categorized into broken/cracked, seeds with hull/husk, spotted seed/discolored seed, insect damage, and undamaged. All individual components were weighed and expressed as a percentage by the weight of the whole component using the formula; (weight of component/total weight) ^*∗*^ 100. The discoloured seeds were grouped into various colours (dark spot, yellowish/pale yellow brown, and greyish white) using a colour chart.

### 2.4. Germination Test

The guidelines of the International Seed Testing Association (ISTA) were followed in the germination test. The experiment involving the top of paper method (TP), involved 100 seeds (25 seeds per plate) replicated 3 times for each sample placed on Petri plates and laid with moist filter paper. The petri dishes were covered with lids. The set up was kept in a germination room under suitable environment (85–90% RH, 25°C). After 10 days, the seeds and seedlings were evaluated and categorized into normal seedlings, abnormal seedlings, hard seeds, fresh seeds, and dead seeds as a percentage of the seeds examined as classified by ISTA. Seedlings that had well developed plant parts, including a well-developed rooting system and well-developed green leaves emerging, were classified as normal seedlings. Seedlings that lacked one of the essential plant part such as a root or leaf or had other defects were classified as abnormal seedlings. Freshly ungerminated seeds were classified as hard seeds. Rotten seeds that failed to germinate were classified as dead seeds.

The germination and composition percentages were calculated based on the average of 100 seeds in 3 replicates and expressed as a percentage using the formula stated below.

(1) $$\begin{array}{c} Germination\;percentage\left(\%\right)=\frac{Total\;number\;of\;germinated\;seeds\;X\;100}{Total\;number\;of\;seeds\;plated}\text{.} \end{array}$$

After the seed germination evaluation, the percentages of the various categories were calculated by the formula;

(2) $$\begin{array}{c} Composition\;percentage\left(\%\right)=\frac{Total\;number\;component\;X\;100}{Total\;number\;of\;seeds\;plated}\text{.} \end{array}$$

### 2.5. Seed Health Test

Two methods (Agar test and Blotter test), were employed for the seed health testing following the International Seed Testing Association [17].

### 2.6. Blotter Test

Hundred seeds each of the various samples were randomly selected and surface sterilized with 2% sodium hypochlorite (NaOCl_2_). Filter papers were moistened and placed in petri plates. One hundred (100) seeds, in batches of 10 seeds, were arranged per plate and replicated 3 times. The seeds in the plates were in incubated for 7–8 days at 25 + 2°C. After the incubation period, pathogens were morphologically identified based on the colony's colour, shape, and size. A compound microscope (Ultra Swift Lite System, China) was then used to examine and confirm the pathogens on the basis of growth of mycelium and other fruiting bodies, spore shape and size as described in the technical bulletin on seed-borne diseases and seed health testing of rice [18]. Unknown cultures were grown on Agar media for improved growth to aid identification.

(3) $$\begin{array}{c} Percentage\;prevalence\;of\;individual\;fungi=\frac{Number\;of\;infected\;seeds\;by\;individual\;fungal\;X\;100}{Total\;number\;of\;seeds\;plated}\text{.} \end{array}$$

### 2.7. Agar Method

Potato Dextrose Agar (PDA) enhanced axenic fungal growth for further identifications after usage of the incubation method. Five (5 g) powdered agar was mixed with 100 ml of water and stirred. It was then autoclaved for 15–20 minutes, after which it was cooled to about 50°C. The Agar was then transferred into the petri-dishes, which solidified in about 20 minutes. Antibiotics and amoxicillin, were added to the Agar to inhibit the growth of bacteria. After the solidification phase, the seeds were surface sterilized with sodium hypochlorite in order to eliminate any form of contamination as well as saprophytic organisms. The saprophytes grow rapidly on the media, which mostly prevents the growth of important pathogens of interest. The seeds were then plated and incubated for 7–8 days at 25 + 2°C. After the incubation period, pathogens were morphologically identified based on the colony's color, shape, and size. A compound microscope (Ultra Swift Lite System, China) was then used to examine and confirm the pathogens on the basis of growth of mycelium and other fruiting bodies, spore shape and size as described in the technical bulletin on seed-borne diseases and seed health testing of rice [18].

### 2.8. Determination of Percentage Incidence of Fungi on Samples Collected from the Three Selected Districts

The seeds infected by fungi were counted for each sample, divided by the total number of seeds plated, and multiplied by 100% to determine the percent incidence.

### 2.9. Fungi Associated with Discolored Seeds

Hundred (100) seeds each from the six varieties were sampled and critically examined. The seeds were grouped according to their colours. The discoloured seeds were grouped into different categories and plated using the blotter method. The different categories were dark spot, brown/dark brown, yellow/pale yellow, and greyish white.

### 2.10. Data Analysis

The IBM SPSS STATICSTICS 22 was used to analyze the field survey data. Fungal pathogen count data were subjected to analysis of variance (ANOVA), and the means were separated using the least significant difference (LSD) at a 5% probability level, in GenStat Statistical Package (12^th^ Edition).

## 3. Results

### 3.1. Farmers' Knowledge and Perception on Fungal Seed-Borne Diseases of Rice, Varieties of Rice Cultivated and Storage Practices Employed

#### 3.1.1. Farmers' Knowledge on Varieties of Rice Cultivated in the Study Area

All the farmers interviewed had knowledge about the varieties of rice they cultivated. They were able to provide the names of the varieties they cultivated. Seven varieties were mostly cultivated by the farmers: AGRA, “Jasmine,” “Lapete,” “Bossom mmienu,” “Mercy,” “AgyaAmoah” and “Kotoko.” The varieties “Bossom mmienu” and “Kotoko” were cultivated by 0.4% of farmers (Table 2). Out of the 230 farmers, 191 representing 83%, cultivated AGRA, followed by the local rice variety, “Lapete,” which was cultivated by twenty-six farmers (11.3%). Five farmers (2.2%) cultivated “Jasmine” rice variety, four farmers cultivated “Agyamoah” rice variety and two farmers cultivated “Mercy.” Only one farmer cultivated each of “Bossom mmienu,” and “Kotoko” rice cultivars.

#### 3.1.2. Source of Seeds for Planting

Forty-three percent (43%) of the respondents planted their saved seeds, 28.3% of the farmers planted certified seeds obtained directly from various MoFA offices (Crops Services Department), and 28.7% used both farmer-saved and certified seeds.

### 3.2. Recognition of Diseases

#### 3.2.1. Rice Blast

One hundred and seventy-nine (179) farmers, representing 77.8% of the respondents, reported that they have observed symptoms of blast at various stages of rice production. However, 51 farmers (22.2%) indicated they did not observe any symptoms of blast on their rice seeds.

#### 3.2.2. Seedling Rot

Seventy-three (73) farmers representing 31.7% of the farmers responded they observed symptoms of seedling rot, however, 157 farmers (68.3%) indicated they did not observe any seedling rot symptoms.

#### 3.2.3. Seedling Blight

Over seventy percent (73.5%) of the farmers had observed seedling blight before, as shown to them on a picture plate. About 26.5% responded that they had not observed any symptoms of seedling blight in their fields.

#### 3.2.4. Respondents Perceptions of the Causes of Diseases

About (25%) of farmers indicated pathogens solely caused diseases on their farms, and 24% indicated both pathogens and soilborne organisms as major causes of diseases. Twenty percent (20%) of the respondents reported soil-borne organisms caused diseases in their farms. However, 9% did not know the causes of diseases in their fields, and 6% reported seed-borne diseases were the causes of diseases on their farms.

#### 3.2.5. Assessment of Physical Quality of Seeds

All seed samples examined had some percentages of undamaged seeds, cracked or broken seeds, and discoloured seeds (Table 3). There were significant differences (*P* < 0.05) among undamaged seeds. The least undamaged seeds (93.1%) were the “Bossome mmienu” seeds. The AGRA FSS rice seed sample collected from Juaboso had the least undamaged (50.21%) seeds.

Seed discolouration was observed on seed samples obtained from farmers as they saved seeds. There were significant differences (*P* < 0.05) among the seed samples regarding seed discolouration. The AGRA rice sample collected as Farmer-saved seed from Juaboso, had the highest level of seed discoulouration (41.96%). Other seed varieties “Lapete” and “Mercy” had seed discolouration values (39.73% and 38%), respectively. There were significant differences (*P* < 0.05) among the seed samples for broken/cracked seeds. The seed sample obtained from MoFA office, Juaboso and NRGP had the least percentage (2.81% and 2.91%) of broken/cracked seeds respectively. The highest percentages (7.91% and 7.83%) of broken seeds were on the “Mercy” rice seed sample and the AGRA rice variety, respectively.

#### 3.2.6. Germination Test and Seedling Evaluation of Rice Samples Collected from Three Districts

The local rice seed sample, “Bossome mmienu,” collected from farmers in Juaboso performed better and had a high germination score (94.67%) of normal seedlings. There were significant (*P* < 0.05) differences among the seed samples with regards to germination percentages (Table 4). The sample with the lowest germination rate was the AGRA rice variety collected from farmers in the Bodi district.

Comparing the AGRA rice varieties, the samples taken from MoFA offices that were certified seeds performed better in terms of germination than the farmer saved seeds. There were significant differences among them. AGRA rice sample collected from Wiawso scored highest among the AGRA rice samples with 93.33% normal seedlings followed by the AGRA rice variety collected from farmers in the Wiawso Municipality (89.67%). The AGRA rice sample collected from farmers in the Wiawso municipal was the only FSS sample which performed better than the MoFA sourced samples. The AGRA rice seed sample collected from farmers in the Bodi district performed worst in terms of germination, scoring 62.33% of normal seedling germination. There were significant (*P* < 0.05) differences among the abnormal seed percentages of the various samples. The AGRA sample collected from the Wiawso Farmers and the Mercy rice seed sample collected from the Juaboso had the lowest percentages (4%) of abnormal seedlings.

### 3.3. Seed Health

#### 3.3.1. Prevalence of Seed-Borne Fungi Using Different Identification Methods

Thirteen fungi were identified, comprising of twelve genera (Table 5). Eight fungi were identified using the blotter method. The Blotter test could not support the growth of some fungi. *Aspergillus niger*, *Aspergillus flavus*, *Fusarium* spp., *Penicillium* spp., *Rhizopus* spp., *Mucor* spp., *Macrophomina phaseolina,* and *Bipolaris oryzae* were identified by both the Agar and Blotter tests. *Pyricularia* spp., *Trichoderma* spp., *Botryodiplodia* spp., *Curvularia* spp., *and Collectitrocum* spp. were identified only through the Agar test.

#### 3.3.2. Fungi Associated with the Rice Seed Samples Collected from the Three Selected Districts

All seed samples collected from the three selected districts had various seed-borne fungi at different levels of infection (Table 6). Thirteen fungi in total were associated with the rice seed samples collected. Six out of the thirteen fungi identified were saprophytic, and seven were pathogenic. Some the saprophytes were *Aspergillus flavus*, *Asperfillus niger*, *Mucor* spp., *Penicillium* spp., *and Rhizopus* spp. All the seed samples collected scored high levels of saprophytes. Not all the fungi identified were present on all the seed samples collected from the different districts. The Juaboso district scored the highest number of fungi identified (Table 6) whiles the Bodi district scored the lowest number of fungi associated with rice seeds. In the Bodi district, *Aspergillus niger* scored the highest frequency of fungi infection (28.25%) followed by *Macrophomina* spp. (23.65%) while *Aspergillus flavus* scored the least frequency of infection with 5.95%.

In the Juaboso District, *Aspergillus flavus* scored the highest frequency of fungi infection at 19.42%, followed by *Aspergillus niger*, 12.38%. *Collectotricum* spp. scored the least frequency of infection at 1.92%. *Aspergillus flavus* scored the highest frequency of infection in the Wiawso District, followed by *Curvularia* spp. (Table 6).

#### 3.3.3. Frequency of Fungi Associated with the Different Rice Seed Varieties Sampled in the Selected Districts

The variety AGRA scored the highest prevalence of fungi followed by the variety “Bossomme mmienu” (Table 7). The variety Mercy scored the least number of fungi infections with only five fungi reported to be associated with the Mercy rice seed sample. All thirteen fungi identified were reported to be associated with the AGRA rice seed variety. *Aspergillus flavus*, *Aspergillus Niger*, *Rhizopus* spp., *and Penicillium* spp. were associated with all the rice seed varieties sampled. The “Mercy” rice variety had the highest prevalence of *Aspergillus niger*, whiles “Agyamoah” rice variety had the highest prevalence rate of *Aspergillus flavus*. *Bipolaris oryzae* was observed on only three varieties: AGRA, “Agyamoah,” and “Bossomme mmienu,” with 13.77%, 9.59%, and 7.36%, respectively. The “Agyamoah” rice variety had the highest prevalence rate of *Bipolaris oryzae. Curvularia* spp. was identified on only two rice varieties*. Pyricularia* spp. was identified on the Kotoko rice variety and the AGRA rice variety at 19.91% and 4.09% frequency of occurrence, respectively (Table 7).

#### 3.3.4. Frequency of Fungi Infection of the Variety AGRA in the Selected Districts

Eleven (11) out of the thirteen fungi identified were observed on the AGRA rice seeds. The Bodi farmer-saved seed sample had the highest number of fungi associated with the seeds however the AGRA Farmer Saved seeds from Wiawso District had the least number of fungi associated with the seeds, mainly saprophytes; *Aspergillus flavus, Aspergillus niger, and Rhizopus* spp. However, this was the only AGRA rice sample which had *Pyricularia* spp. associated with it (Table 8).

#### 3.3.5. Discolored Seeds and Associated Fungi

Fungi cause seed discolouration in varying forms. Fungi which had dark brown/brown discolouration of rice seeds were *Bipolaris oryzae, Fusarium* spp.*, Macrophomina* spp. and *Aspergillus* spp. (Table 9). However, only *Bipolaris oryzae* was associated with the yellow/pale yellow colour. *Bipolaris oryzae, Curvularia* spp., *Fusarium* spp., and *Botryodiplodia* spp., were associated with the dark spot discolouration. *Alternaria* spp. and *Aspergillus* spp. were identified on the greyish white seed discolouration sample.

## 4. Discussion

### 4.1. Knowledge of Farmers on Varieties of Seeds Cultivated

Majority of the farmers interviewed planted Agra rice followed by Lapete, a local rice variety. “Bossom mmienu” and “Kotoko” varieties were cultivated by only 0.4% of the respondent. AGRA rice was the mostly used variety by the farmers constituting 83%. This is as a result of various Programs and projects enrolled by the Government of Ghana through the Ministry of Food and Agriculture and other institutions such as Ghana Rice Interprofessional Body. In a previous study by Ragasa et al. [19], increased use of certified seeds was encouraged through projects initiated by the Government of Ghana and or Development Agencies. The Planting for Food and Jobs under MOFA was a major flagship program introduced by the Government of Ghana in 2017 to promote the use of the AGRA rice variety. In spite of all these interventions, some farmers still preferred to use local farmer-saved seeds. A rice variety “Agya Amoah” which was introduced to the farmers in the selected districts back in 1987 was still in use by some farmers. The farmers indicated that, even though there were programs and projects to enhance the use of certified seeds and improved varieties, the local varieties, for example, Lapete was also an early maturing variety and resistant to pest and diseases. They explained that the local varieties were able to withstand harsh weather conditions such as drought, pests and flooding compared to the newly improved varieties, even though the improved varieties give higher yields. In a study by Marfo et al. [20] rice varieties such as “AgyaAmoah,” “Mercy,” “Bosome Mmiensa,” “Bosome Nnan,” “Kotoko,” “Abankora,” and “Sikamo” were grown by farmers in the Western Region. However, the preferred variety was “Agya Amoah” because of the following characteristics: yield (45%), grain size (17%), and early maturity (16%).

### 4.2. Source of Seeds for Planting

Majority of the farmers interviewed (70%) use their saved seeds for planting in the next season, and about 28% of them used certified seeds. The farmers used their own saved seeds because, they did not have access to certified seeds easily, some also preferred to use their saved seeds because of lower costs. Some farmers however indicated there were no differences between their saved seeds and the certified seeds. Those who used certified seeds obtained their seeds from the district agriculture offices, few agro input shops, and NGOs. The percentage of farmers (28%) using certified seeds relatively increased compared to percent of farmers listed in the literature study (11%) [19]. This was because certified seeds were obtained from MoFA under the Planting for Food and Jobs (PFJ). Farmer-saved seeds were the main source of seeds for the farmers in the three districts. Over 70% of rice farmers depend on their saved seeds for subsequent seasons, especially in the sub-Saharan Africa where most farmers do not buy certified seeds, because of high prices [21]. Those who used farmer-saved seeds and local varieties acquired their seeds in the form of seed exchange, gifts, and from the local market.

### 4.3. Rice Seed Storage and Storage Practices of Farmers in the Three Selected Districts

Majority of the farmers (96.1%) in the three districts stored their seeds at home under ambient temperatures in nylon bags and jute sacks. About 2.6% of the farmers stored their seeds in a warehouse operated either by a private individual or MoFA, and 1.3% of the respondents stored their seeds in cold storage. Most farmers in developing countries, e.g., Ghana, store their seeds under ambient conditions where temperature and moisture content vary.

The 1.3% of farmers who stored their seeds in cold storage explained they were advised by some agricultural officers to do so. They usually stored seeds of new varieties introduced to them by agricultural officers. They stored their seeds in their home refrigerators usually only for a season at temperatures below 10°C. The warehouse storage was managed by a private individual who stored assorted products for a fee payable monthly. They were usually rice mill operators who stored farmers' seeds for them. The majority of farmers who stored their seeds in nylon and jute sacks followed the traditional practice of storing produce. They stored them either in the store rooms or kitchens without measuring temperature and relative humidity (RH) of the storage environments. The practice of seed storage in these locations may affect the seed quality and increase the development of pathogens on the rice seeds. The bags for storing seeds may not be properly sealed allowing seeds to be exposed to moisture and other environmental factors, which enhance growth of microorganisms on the seeds. Rice with a high moisture content >21% increases rice discoloration from temperatures 10°C to 40°C as storage period progresses. There is a higher expression of discoloration patterns on rice seeds stored at the high temperatures for e.g., 40°C compared to storage at lower temperatures [22]. Most rice farmers in Ghana lack moisture meters to determine the moisture content of their seeds and this together with storage environments with high temperatures >10°C results in mold development and discoloration of their seeds. In the study by [22] the total discoloured kernel area increased with increasing storage moisture content (MC), temperature, and storage duration, with about 100% discolouration observed at 16 weeks at 40°C storage temperature.

Over 98% of the farmers did not treat their seeds (farmer-saved or certified) before storage. Farmers who did not treat their seeds said, thorough drying under the sun enhanced the prolonged storage of their seeds. Those who treated their seeds before storage used various insecticides such as Akate Master and Lambda. These insecticides were used because they believed any poisonous chemical will be effective in controlling any kind of infection or pathogen. The chemicals used by the farmers for treating their seeds were also used on other crops, for e.g., cocoa. Rice seed coating with for example *Trichoderma* spp., has been found to be very useful in maintaining the seed quality and tolerance to biotic and abiotic stress [23].

Most of the farmers did not really observe changes associated with their seeds during storage. They did not bother to check for these changes until planting. However, 61% of farmers in the Ashanti region of Ghana observed changes associated with rice seeds during storage, they usually observed pest infestation during storage as reported [13].

Most of the farmers stored their seeds for three to five months. Only few of them stored up to 12 months. Farmers in the three districts did not store their seeds beyond one year. Inadequate storage facility inhibited storage for relatively longer periods. Most of farmers did all year-round farming (two to four times a year), therefore will need seeds for planting frequently. In the Ashanti region, 48% of rice farmers stored their seeds beyond six months [13]. Those who stored their seeds for longer periods were likely to attract more pathogens. Depending on the storage condition, there quality of seeds reduced with increasing storage duration [24].

### 4.4. Farmers Perception of Occurrence of Some Important Rice Diseases

Farmers had little knowledge about seed-borne diseases during a focal group discussion session. An average of about 60% of the farmers reported to have observed various symptoms of the seed-borne diseases. The majority of the farmers (77.8%) observed symptoms of blast at various stages; 73.5% observed seedling blight whiles only 30% observed seedling rot. In a previous study, the majority of farmers interviewed in the Greater Mwea region of Kenya were aware of the occurrence of rice diseases for e.g., rice blast disease [25]. From the survey conducted, farmers ignored symptoms of the various seed-borne diseases when these appeared during storage or on the fields. Those who observed the symptoms of diseases perceived various causes of the diseases. About 50% of the farmer-perceived diseases were caused by pathogens, and few of them attributed the causes of the diseases to the source of seeds, soil, and insects. Farmers in some selected districts in Ghana paid much attention to the symptoms of diseases. The farmers made observations of symptoms of seed-borne diseases such as rice blast, and they associated the symptoms with being caused by pathogens [15]. Similarly, rice farmers in Burkina reported diseases as a major constraint to rice production and were aware of the causes of the diseases [26].

### 4.5. Knowledge of Seed Borne Diseases

Majority of the respondents (87%) had observed the incidence of seed-borne diseases. They responded that they were aware of the fact that diseases “sit in the seeds.” Thirteen (13%) of the respondents did not have any knowledge on seed-borne diseases. In one study by Kihoro et al. [25] in Kenya, they reported that 98% of rice farmers had knowledge about rice blast, a seed-borne disease.

Out of those who had knowledge about seed-borne diseases, 50% treated their seeds with different kinds of chemicals before planting. Those who treated their seeds used any chemical they perceived to be poisonous for e.g., Akate Master, Lamda, and Dusband. Those who did not treat their seeds before planting relied on the sun as a treatment to control seed-borne diseases [13]. Rice farmers in Bangladesh, treated their seeds with hot water, as well as selected fungicides (Bavistin 50 WP, Dithane M 45 and neem extract) [27].

### 4.6. Detection and Identification of Seed-Borne Fungi on the Rice Samples Collected from the Three Selected Districts

The current study identified thirteen fungi to be associated with the rice seeds (Table 5). In a previous study, rice seeds were associated with 20 fungi in the Ashanti Region. Among these, the following were not identified in the current study, there were *Alternaria padwickii, Alternaria* spp*.,, Cercospora* spp*., Curvularia lunata, Curvularia pallescens, Fusarium verticillioides, F. oxysporum, F. pallidoroseum, F. solani, Nigrospora oryzae, Phoma* spp*., Sarocladium oryzae,* and *Stemphylium* spp*. Trichoderma* spp., *Macrophomina phaseolina*, *Botrydiplodia* spp., and *Pyricularia* spp. identified in our isolations were absent in those of Asamoah [13]. Similar fungi as identified in the current study have been identified on rice in Ghana [15, 28, 29]. Mucor spp. identified in this study, was recently isolated from rice seeds in Kenya [27]. In another study, Nustugah et al. [15] identified *Bipolaris oryzae, Pyricularia* spp. and *Magnaporthe* spp. to be present in Ghana in the northern region and upper east regions of Ghana. The absence of *Magnaporthe oryzae* from our study sites, confirms the absence of rice blast disease caused by these fungi in farmers' fields in the current study. Rice seed-borne fungi such as *Bipolaris oryzae, Cercospora* sp.*, Colletotrichum* sp., *Curvularia lunata,* and *Curvularia pallescens* have been reported [30, 31]. The high prevalence of storage fungi such as *Aspergillus flavus* and *Aspergillus niger* on all rice seed samples was an indication that the seeds were stored under high temperature, and had high moisture content. High temperature and moisture at storage have been found to decrease germination of seeds of soy and maize as well as increasing the infection of *Aspergillus flavus*. The high prevalence of storage fungi such as *Aspergillus* spp. have been reported to be associated with high moisture content [32]. The high prevalence of storage fungi may also cause seed rotting. Storage fungi such as *Aspergillus* spp. and *Fusarium* spp. not only enhance discolouration of the rice kennel but also secrete deadly mycotoxins detrimental to the human and animal health.

In a previous study, the fungi species *Alternaria* spp.*, Bipolaris* spp., and *Fusarium* spp. were associated with various discolouration on rice [33]. In their isolations, *Alternaria padwickii* was responsible for the ash gray discolouration, *Fusarium* spp. was responsible for light pink and pale yellow discolouration. In the current study, *Alternaria* spp. and *Fusarium* spp. were responsible for grayish white discolouration. *Bipolaris oryzae* was associated with dark brown, brown spot, and brown discolouration.

*Bipolaris* spp. has been found to be the most important pathogen of rice and the most frequently identified pathogen on rice seeds [27]. This organism has been reported to be the cause of brown spot and seedling blight diseases of rice. *Pyricularia* spp. has also been reported to be one of the principal causal organisms in rice blast. Both of these two important seed-borne fungi (pathogens) were identified in the current study.

## 5. Conclusions

This study showed that seed-borne fungi were associated with all the rice cultivars sampled from the study area. The fungi were made up of six saprophytes and seven pathogens. There was high prevalence of storage fungi associated with the rice seeds partly because of the storage practices employed by the farmers. There is therefore the need to elucidate the biochemical basis of rice discoloration and the relationship with specific microorganisms. This will enable development of microbe-specific interventions against rice discolouration.

## Figures and Tables

**Table 1:** Sources and varieties of seed samples obtained from the three selected districts.

District	Community	Variety	Source
Wiawso municipal	Datano, Boako, Amprompe	AGRA rice	Farmer saved seed
	MoFA district office	AGRA rice	Certified
	Asafo (NRGP)	AGRA rice	Local seed grower

Juaboso	Juaboso, Nkwanta, Bonsu	AGRA rice	Farmer saved seed
	Benchemaa, Nkatieso, Proso-Kofi krom	Kotoko	Farmer saved seed
	Nkatieso, Proso-Kofi Krom	Agyamoah	Farmer saved seed
	Proso-Kofi krom	Mercy	Farmer saved seed
	Proso-Kofi krom	Bossome mmienu	Farmer saved seed
	MoFA district office	AGRA rice	Certified

Bodi	Afere, Aferewaa Amoaya	AGRA rice	Farmer saved seed
	Aferewaa	Lapete	Farmer saved seed
	MoFA district office	AGRA rice	Certified

**Table 2:** Percent distribution of varieties of rice cultivated by farmers in the three rice growing districts.

Varieties of rice	Number of farmers	Percentage
AGRA	191	83.0
Lapete	26	11.3
Jasmine	5	2.2
Agyamoah	4	1.7
Mercy	2	0.9
Bossom mmienu	1	0.4
Kotoko	1	0.4
Total	230	100.0

**Table 3:** Categories of seed types and their percentages collected from three districts.

Source	Sample	Undamaged	Damaged		Total
			Cracked/broken	Discoulored	
Certified (MOFA)	Agra Wiawso	58.20	7.82	33.98	100.00
	Agra Juaboso	78.31	2.81	18.88	100.00
	AGRA BODI Mofa	81.08	3.12	15.80	100.00

Local seed producer	Agra Nrgp	58.13	2.91	38.96	100.00

Farmer saved seeds (FSS)	Agra Wiawso Fss	68.10	4.17	27.73	100.00
	Agra Juaboso Fss	50.21	7.83	41.96	100.00
	Agra Bodi Fss	62.31	3.89	33.80	100.00
	Mercy Juaboso	54.09	7.91	38.00	100.00
	Kotoko Juaboso	89.06	2.98	7.96	100.00
	Agyamoah Juaboso	71.32	4.01	24.67	100.00
	“Bosomme Mmienu”	93.12	3.58	3.30	100.00
	Lapete Bodi	55.89	4.38	39.73	100.00

	LSD	4.09	0.44	2.24	
	*P* < 0.05	0.001	0.001	0.001	

**Table 4:** Percent categorization of the various components of the evaluation of germination test into normal, abnormal, fresh, and dead seeds in the three selected districts.

Source	Seed sample	Normal seedlings	Abnormal seedlings	Fresh seeds	Dead seeds
Certified (MOFA)	Agra Wiawso	93.33	4.27	1.20	1.20
	Agra Juaboso	83.00	10.27	0.00	6.73
	Agra Bodi	81.33	11.93	2.20	4.54

Local seed producer	Agra	75.00	9.83	2.73	12.44

Farmer saved seeds	Agra wiawso	89.67	4.00	2.00	4.33
	Agra Juaboso	74.00	13.33	3.63	9.04
	Agra Bodi	62.33	5.83	4.83	27.01
	Mercy Juaboso	91.33	4.00	1.87	2.80
	Kotoko Juaboso	89.00	5.00	0.00	6.00
	Agyamoah Juaboso	80.67	9.20	2.80	7.33
	“Bosomme Mmienu”	94.67	1.67	0.70	2.96
	Lapete Bodi	80.67	11.2	0.33	7.80

	LSD	7.59	1.17	1.17	1.32
	*P* < 0.05	0.001	0.001	0.001	0.001

**Table 5:** Fungi isolated and identified using different methods (blotter and agar).

Fungi identified	Blotter	Agar
*Aspergillus Niger*	^*∗∗*^	^*∗∗*^
*Aspergillus flavus*	^*∗∗*^	^*∗∗*^
*Fusarium* spp.	^*∗*^	^*∗∗*^
*Trichoderma* spp.	−	^*∗*^
*Macrophomina phaseolina*	^*∗*^	^*∗*^
*Rhizopus* spp.	^*∗*^	^*∗∗*^
*Bipolaris oryzae*	^*∗*^	^*∗∗*^
*Botryodiplodia* spp.	−	^*∗*^
*Curvularia* spp.	−	^*∗*^
*Pyricularia* spp.	−	^*∗*^
*Penicillium* spp.	^*∗∗*^	^*∗∗*^
*Collectotricum* spp.	−	^*∗*^
*Mucor* spp.	−	^*∗*^

^*∗∗*^Frequency above 50%, ^*∗*^frequency below 50%, —fungi not observed.

**Table 6:** Frequency of fungi on rice seeds samples collected from the three selected districts.

Fungi identified	Bodi	Juaboso	Wiawso
*Aspergillus Niger*	++	++	++
*Aspergillus flavus*	+	++	++
*Fusarium* spp.	++	+	++
*Trichoderma* spp.	+	+	−
*Macrophomina phaseolina*	++	+	+
*Rhizopus* spp.	++	+	++
*Bipolaris oryzae*	−	+	+
*Botryodiplodia* spp	−	+	−
*Curvularia* spp	−	++	++
*Pyricularia* spp	+	+	++
*Penicillium* spp	+	+	+
*Collectotricum* spp	−	+	−
*Mucor* spp	−	+	+

− = not present, + = incidence below 10%, ++ = incidence between 11–30%, +++ = incidence above 30%. NB: + = low prevalence, ++ = moderate prevalence, +++ = high prevalence.

**Table 7:** Fungi identified on rice seed varieties collected from the three selected districts tested by the blotter method.

	Agra	Lapete	Mercy	Bossome Mmienu	Agyamoah	Kotoko
*Aspergillus Niger*	+++	+++	+++	+++	+++	+++
*Aspergillus flavus*	++	+	+	+	+++	++
*Fusarium* spp.	++	++	++	++	−	++
*Trichoderma* spp	+	−	−	+	−	+
*Macrophomina phaseolina*	*++*	−	−	*+*	*+*	−
*Rhizopus* spp.	++	+++	++	+	++	+
*Bipolaris oryzae*	++	−	−	+	+++	−
*Botryodiplodia* spp	++	−	−	−	−	−
*Curvularia* spp	++	−	−	−	+++	−
*Pyricularia* spp	+	++	−	−	++	++
*Penicillium* spp	+	+	+	+	+	+
*Collectotricum* spp	+	−	−	−	−	−
*Mucor* spp	+	−	+	−	+	−

− = not present, + = incidence below 10%, ++ = incidence between 11–30%, +++ = incidence above 30%. NB: + = low prevalence, ++ = moderate prevalence, +++ = high prevalence.

**Table 8:** Comparison of the fungal infection of samples of the variety AGRA collected from different sources (MoFA, farmer-saved seeds (FSS) and NRGP) in the three selected district.

	Agra Wiawso MoFA	Agra Wiawso FSS	Agra Wiawso NRGP	Agra Juaboso MOFA	Agra Juaboso FSS	Agra Bodi FSS	Agra Bodi MOFA
*Aspergillus Niger*	++	+++	++	++	++	+++	+
*Aspergillus flavus*	+++	+	+++	+++	+++	+++	+++
*Fusarium* spp.	++	−	++	−	++	++	++
*Trichoderma* spp.	−	−	−	−	−	++	+
*Macrophomina phaseolina*	++	−	++	−	−	++	−
*Rhizopus* spp	++	++	++	++	−	++	++
*Bipolaris oryzae*	++	−	+++	−	++	++	−
*Botryodiplodia* spp	++	−	++	−	−	++	−
*Curvularia* spp	+++	−	−	−	+	+++	−
*Pyricularia* spp.	−	+	−	−	−	−	−
*Penicillium* spp	−		−	−	−	−	−
*Collectotricum* spp	−	−	−	−	−	−	−
*Mucor* spp	−	−	+	−	+	−	−

− = not present, + = incidence below 10%, ++ = incidence between 11–30%, +++ = incidence above 30%. NB: + = low prevalence, ++ = moderate prevalence, +++ = high prevalence.

**Table 9:** Percentage of seed-borne fungi associated with various discolouration types in the rice seed samples collected from the three selected districts.

Type of discolouration	Pathogens associated						
	*Bipolaris oryzae*	*Curvularia* spp.	*Fusarium* spp.	*Alternaria* spp.	*Macrophomina* spp.	*Botryodiplodia* spp.	*Aspergillus* spp.
*Dark brown/brown*							
Agra	39	0	41	0	7	0	18
Agyamoah	48	0	33	0	5	0	5
Kotoko	12	0	19	0	9	0	9
Mercy	29	0	9	0	18	0	11
Lapete	31	0	18	0	12	0	3
Bossom Mmienu	5	0	41	0	15	0	3

*Yellow/pale yellow*							
Agra	31	0	0	0	0	0	0
Agyamoah	28	0	0	0	0	0	0
Kotoko	5	0	0	0	0	0	0
Mercy	11	0	0	0	0	0	0
Lapete	9	0	0	0	0	0	0
Bossom Mmienu	5	0	0	0	0	0	0

*Dark spot*							
Agra	19	27	11	0	0	5	0
Agyamoah	21	8	8	0	0	8	0
Kotoko	8	9	10	0	0	11	0
Mercy	11	4	0	0	0	19	0
Lapete	9	18	0	0	0	8	0
Bossom Mmienu	6	3	0	0	0	7	0

*Greyish white*							
Agra	0	0	5	16	0	0	0
Agyamoah	0	0	8	21	0	0	3
Kotoko	0	0	6	9	0	0	2
Mercy	0	0	3	11	0	0	3
Lapete	0	0	11	18	0	0	1
Bossom Mmienu	0	0	8	5	0	0	0

## Data Availability

The authors confirm that the data supporting the findings of this study are available within the article.

## Supplementary Materials

Supplementary Materials Data survey sheet included under the Supplementary Method.

## References

[1] USDA (2018). World Agricultural Production. https://www.fas.usda.gov/data/world-agricultural-production.

[2] FAO (2020). *Food and Agriculture Organization of the United Nations*.

[3] Adomako K. W., Yun T. H., Appiah-Twum F., Akolgo I. G., Asamoah E. O. (2020). Factors influencing the production of local rice in Ghana: the moderating role of open innovation. *International Journal of Scientific Research in Science, Engineering and Technology*.

[4] Nanda J. S., Sharma S. D. (2012). Crops that feed the world 7: rice. *Food Security*.

[5] Ziska L. H., Gealy D. R., Burgos N. (2015). Weedy (red) rice: an emerging constraint to global rice production. *Advances in Agronomy*.

[6] Reddy O. R., Sathyanarayana N. (2001). Seed-borne fungi of rice and quarantine significance. *Major Fungal Diseases of Rice*.

[7] Singh K. M., Jha A., Meena M., Singh R. (2012). Constraints of rainfed rice production in India: an overview. *in Innovations in Rice Production, Eds: P. K. Shetty, M. R. Hegde, and M. Mahadevappa*.

[8] Strange R. N., Scott P. R. (2005). Plant disease: a threat to global food security. *Annual review of phytopathology*.

[9] Butt A. R., Yaseen S. I., Javaid A. (2011). Seed-borne mycoflora of stored rice grains and its chemical control. *Journal of Animal and Plant Sciences*.

[10] Akhtar N., Mirza J. H., Bajwa R., Javaid A. (2007). Fungi associated with Seeds of some economically important plants. *Mycopath*.

[11] Ou S. H. (1985). *Rice Diseases*.

[12] Ebrahim Zainali S. M., Zainali E., Ghaderi-Far F., Soltani E., Chaleshtari M. H., Khoshkdaman M. (2014). Evaluation seed-born fungi of rice [oryza sativa L.] and that effect on seed quality. *Journal of Plant Pathology & Microbiology*.

[13] Asamoah J. F. (2012). *Seed-Borne Fungi of Farmer-Saved Rice (Oryza sativa L.) Seeds from Inland Valleys of Three Districts in Ashanti Region of Ghana and Their Management*.

[14] Sadia O. A. (2012). Seed health testing of rice and the comparison of field incidence and laboratory counts of *Drechslera oryzae* (*Bipolaris oryzae*) and *Pyricularia oryzae* in Ghana.

[15] Nutsugah S., Dogbe W., Twumasi J. (2005). Prevalence of rice blast and varietal screening in Ghana. *Journal of Science and Technology*.

[16] West Africa Centre for Crop Improvement (WACCI) (2023). Francis Mensah Ackaah. https://wacci.ug.edu.gh/content/francis-mensah-ackaah.

[17] ISTA (2023). * Seed quality Assurance, “International rules for seed testing”*.

[18] Agarwal P. C., Mortensen C. N., Mathur S. B. (1989). *Seed-Borne Diseases and Seed Health Testing of Rice. Technical Bulletin No. 3, Phytopathological Papers No. 30*.

[19] Ragasa C., Dankyi A., Acheampong P. (2013). Patterns of adoption of improved rice technologies in Ghana. *Ghana Strategy Support Program (GSSP) Working Paper*.

[20] Marfo K. A., Dorward P. T., Craufurd P. Q., Ansere-Bioh F., Haleegoah J., Bam R. (2008). Identifying seed uptake pathways: the spread of Agya Amoah rice cultivar in southwestern Ghana. *Experimental Agriculture*.

[21] Samberg L. H., Shennan C., Zavaleta E. (2013). Farmer seed exchange and crop diversity in a changing agricultural landscape in the southern highlands of Ethiopia. *Human Ecology*.

[22] Shafiekhani S., Wilson S. A., Atungulu G. G. (2018). Impacts of storage temperature and rice moisture content on color characteristics of rice from fields with different disease management practices. *Journal of Stored Products Research*.

[23] Cortés-Rojas D., Beltrán-Acosta C., Zapata-Narvaez Y. (2021). Seed coating as a delivery system for the endophyte *Trichoderma koningiopsis* Th003 in rice (*Oryza sativa*). *Applied Microbiology and Biotechnology*.

[24] Tang E., Ngome A. (2015). Rice seed quality as influenced by storage duration and package type in Cameroon. *International Journal of Brain and Cognitive Sciences*.

[25] Kihoro J., Bosco N. J., Murage H., Ateka E., Makihara D. (2013). Investigating the impact of rice blast disease on the livelihood of the local farmers in greater Mwea region of Kenya. *SpringerPlus*.

[26] Traoré V. S. E., Néya B. J., Camara M., Gracen V., Offei S. K., Traoré O. (2015). Farmers’ perception and impact of rice yellow mottle disease on rice yields in Burkina Faso. *Agricultural Sciences*.

[27] Nganga E. M., Kyallo M., Orwa P. (2022). Foliar diseases and the associated fungi in rice cultivated in Kenya. *Plants*.

[28] Nutsugah S., Vibeke L., Atokple I. D. K., Ayensu F. K. (2004). Seed-borne mycoflora of major food crops in Ghana. *Journal of Science and Technology*.

[29] Sreenivasaprasad S., Johnson R. (2013). *Major Fungal Diseases of Rice: Recent Advances*.

[30] Javaid M. S., Wahid A., Idrees M., Gill M. A., Saleem A. (2002). Seed mycoflora studies in rice. *Pakistan Journal of Phytopathology*.

[31] Signaboubo S., Noumbo G. R., Roger K. J. (2016). Seed-borne fungi associated with rice seeds varieties in biognqgor, Chad republic. *International Journal of Current Microbiology and Applied Sciences*.

[32] Niaz I., Dawar S., Sitara U. (2011). Effect of different moisture and storage temperature on seed borne mycoflora of maize. *Pakistan Journal of Botany*.

[33] Ibrahim E. A. M., El-Dahab M. S. A. (2014). Seed discoloration and their effect on seedlings growth of Egyptian hybrid rice. *Research Journal of Seed Science*.

[34] Traoré V. S., Néya B. J., Camara M., Gracen V., Offei S. K., Traoré O. (2015). Farmers’ perception and Impact of rice yellow mottle disease on rice yields in Burkina Faso. *Agricultural Sciences*.

