# Supplementary material for: Seed-Borne Fungi Associated with Diverse Rice Varieties Cultivated in the Western North Region of Ghana
Source: Int J Microbiol. 2023 Jan 27;2023:8690464. doi: 10.1155/2023/8690464 (PMC9897915; doi:10.1155/2023/8690464)
Supplement: Supplementary Materials — Data survey sheet included under the Supplementary Method. [file 8690464.f1.docx]

**Supplementary Method**

**Seed-borne fungi associated with diverse rice varieties cultivated in the Western North Region of Ghana**

***Please do well to circle, rank and mark the corresponding answers to the questions when applicable.***

QUESTIONNAIRE NO…………. DATE……………....

LOCATION…………………………………….

CONTACT OF RESPONDENT: ………………………........

**SECTION A**

**DEMOGRAPHIC DATA**

1. Name: ……………………………………………
2. Age (in years): ……………………………………………
3. Gender: A. Male B. Female
4. Marital status: A. Single B. Married C. Divorced

5. Religion:

A. Islamic B. Christianity C. Traditionalist

Others (specify)….……………...................

6. Level of education: A. No School B. Primary C. Secondary/Vocational

D. Tertiary E. Adult literacy

**SECTION B**

**PRODUCTION**

7. How long have you been in rice production? ………………….. Years

8. How long have you been cultivating rice on your current land? ................................ Years

9. What varieties do you grow and why?

………………………………………………………………………………………………………………………………………………………………………………………………………………………………………………………………………………………………………………………

10. Source of seeds: A. farmer saved . B. Certified seeds C. Both

If A or C,

11. How do you store your harvested rice seeds? A. Warehouse B. Cold Room

C. Farmers’ Room

12. Do you treat your seeds before storage? A. No B. Yes

If Yes,

13. What treatment do you apply before rice seed storage?

14. Do you observe any symptoms of contamination during seed storage? A. No B. Yes

15. What are the symptoms observed on the rice seeds during storage?

………………………………………………………………………………………………………………………………………………………………………………………………………………………………………………………………………………………………………………………

16. How long do you store your rice seeds before planting? ……………… months

17. What are the main symptoms encountered in your rice production system? (Show a picture plate to farmers)

A. B. C. D.

18. At what stage of production do you observe 'A' above? A. Seeds B. Seedlings C. vegetative stage D. Flowering E. Maturity

19. At what stage of production do you observe 'B' above? A. Seeds B. Seedlings C. vegetative stage D. Flowering E. Maturity

20. At what stage of production do you observe 'C' above? A. Seeds B. Seedlings C. vegetative stage D. Flowering E. Maturity

21. At what stage of production do you observe 'D' above? A. Seeds B. Seedlings C. vegetative stage D. Flowering E. Maturity

22. What do you know are the causes of these diseases?

………………………………………………………………………………………………………………………………………………………………………………………………………………

23. How do you manage or control these diseases? ………………………………………………………………………………………………………………………………………………………………………………………………………………………………………………………………………………………………………………………

24. Do you know that some of the diseases are seed-borne? A. No B. Yes

25. Do you treat your seeds before sowing? A. No B. Yes

If yes,

26. What chemical do you use to treat your seeds before planting?

………………………………………………………………………………………………………………………………………………………………………………………………………………

27. How do you select your varieties for planting? ..........................................................................

………………………………………………………………………………………………………

28. Do you plant under irrigation or rain-fed? ……………………………………………………..

29. At maturity, how do you determine your seeds are due for harvesting?

…………………………………………………………………………………………………………………………………………………………………………………………………………

30. How do you harvest your matured rice grain/seed?

……………………………………………………………………………………………………………………………………………………………………………………………………………..

31. How do you process your seeds if you use your own seeds?

………………………………………………………………………………………………………………………………………………………………………………………………………………

Remarks

………………………………………………………………………………………………………………………………………………………………………………………………………………………………………………………………………………………………………………………
